# Prevalence of *Helicobacter pylori* Infection and Micronutrient Deficiencies in a Clinically Referred Cohort of Ezidi Refugees in Rural Armidale: Findings from a Retrospective Study

**DOI:** 10.1007/s10903-025-01715-9

**Published:** 2025-07-01

**Authors:** Grace Noh, Nelson Tran, Oliver McMorran, Edric Hu, Joëlle V. F. Coumans, Salma Hago Mustafa Ali

**Affiliations:** University of New England School of Rural Medicine, Armidale, Australia

**Keywords:** *Helicobacter pylori*, Ezidi refugees, Vitamin B12 and D, Iron

## Abstract

In Australia, Middle Eastern refugees, notably the Ezidi community (an ethnic and religious minority from Northern Iraq and Syria) are disproportionately affected by high rates of vitamin and iron deficiencies. These deficiencies pose significant health risks and can impact overall well-being. Recent studies have suggested a possible correlation between *Helicobacter pylori* (*H. pylori*) infection and deficiencies in essential vitamins and iron, highlighting an important area of research that warrants further exploration. Understanding the relationship between H. pylori infection and nutrient deficiencies in newly arrived Ezidi refugees is critical for several reasons. First, it may provide insights into the underlying health challenges faced by this population, who often arrive with pre-existing health issues. Second, establishing this association could inform current screening practices, allowing for targeted interventions that address both H. pylori infection and nutritional deficiencies. Ultimately, this research aims to contribute to the development of effective health strategies that enhance the well-being of Ezidi refugees, ensuring they receive the necessary support to thrive in their new environment. By focusing on this intersection of infectious disease and nutritional health, we can better understand and mitigate the risks faced by this vulnerable group. A retrospective cohort study was performed using data collected from Ezidi refugees in Armidale Medical Centre and Armidale Hospital from 2018 to 2024. The data was analysed to determine the prevalence of *H. pylori* and deficiencies in iron, vitamin B12, and vitamin D. Further analysis was done with data from Armidale Medical Center only, to study any potential association between *H. pylori* infection and those same micronutrient deficiencies. *H. pylori* infection was detected in 76.7% of refugees tested. The rates of iron deficiency were 35.2% at Armidale Medical Centre and 41.9% at Armidale Hospital. Vitamin B12 deficiency was found in 50.7% and 58% of individuals, and vitamin D deficiency was present in 79.6% and 82.6%, respectively. No statistically significant associations were found between *H. pylori* infection and iron or vitamin D deficiency. However, a statistically significant association was observed between the absence of *H. pylori* and vitamin B12 deficiency. There is a significantly higher prevalence of *H. pylori* infection, iron deficiency, vitamin B12 deficiency, and vitamin D deficiency among Ezidi refugees in Armidale. The relationship between *H. pylori* infection and micronutrient deficiencies remains unclear. Further research is needed to clarify these associations and guide future screening protocols for Ezidi refugees.

## Background

*Helicobacter pylori* (*H. pylori*) is a gram-negative, rod-shaped bacteria that infects the stomach’s lining (1) and is one of the most common bacterial infections globally. *H. pylori* infection is associated with several gastrointestinal diseases, including chronic gastritis, gastric and duodenal ulcers, and in more severe cases, gastric cancer or mucosa-associated lymphoid tissue (MALT) lymphoma(2). Studies have demonstrated that *H. pylori* eradication can lower the incidence of gastric cancer (2, 3).

*Helicobacter pylori* infections have also been associated with micronutrient deficiencies, especially in populations with pre-existing health disparities like refugees. Refugees face a higher risk of malnutrition and micronutrient deficiencies due to factors such as inadequate access to food, displacement, acculturation challenges, and limited access to healthcare(4, 5). Middle Eastern countries have a higher incidence of vitamin B12, vitamin D, and iron deficiencies(6). Given the high prevalence of these deficiencies among Middle Eastern refugees in Australia, a rate notably higher than the estimated prevalence of iron deficiency in the Australian population, which affects approximately 3.3% of adults (7), it is reasonable to assume that they might also affect Ezidi individuals, a subgroup of refugees from Iraq and Syria.

The biological mechanisms behind micronutrient deficiencies are not fully understood but several potential factors have been proposed. These include intestinal bleeding, atrophic gastritis, hepcidin upregulation, and long-term autoimmune changes(8, 9). A recent meta-analysis (10) suggested negative effects of *H. pylori* infection on serum vitamin B12, folate, vitamin C, and vitamin D levels. While the impact of *H. pylori* infection on serum vitamin D levels remains unclear, there is evidence that vitamin D supplementation can positively influence the efficacy of *H. pylori* eradication therapy(9, 11, 12). Systematic reviews have found a significant association between *H. pylori* infection and depleted iron stores, as well as improvements in serum ferritin and haemoglobin levels following *H. pylori* eradication therapy(13, 14).

Australia has one of the highest intake rates of refugee or refugee-like backgrounds. The prevalence of *H. pylori* infection in refugees in Australia is 1.5 times higher than in the general Australian population(15). Current Australian guidelines recommend that all refugees undergo a comprehensive health assessment within 12 months of arrival, including screening for anaemia, vitamin B12, and vitamin D deficiency(16), *H. pylori* screening is not routinely performed unless symptoms are present (e.g., dyspepsia or peptic ulcer disease) or presence of a family history of gastric cancer(16, 17). This approach aims to reduce overtreatment and address concerns about decreased treatment efficacy due to antibiotic resistance(18). However, *H. pylori* testing is recommended in cases of unexplained iron deficiency anaemia and vitamin B12 deficiency(17).

## Theoretical/Conceptual Framework

Given that *H. pylori* disrupts gastric function, and potentially impairs nutrient absorption, (e.g., iron, vitamin B12, and D), and that refugees, especially those from conflict-ridden regions, face a higher risk of malnutrition due to limited access to nutritious food and healthcare, there is a need to examine the role of *H. pylori* in exacerbating these micronutrient deficiencies. Current Australian health guidelines do not mandate routine screening for *H. pylori* in asymptomatic refugees, potentially overlooking a key factor to malnutrition in this vulnerable population. Therefore, this study aims to clarify the role of *H. pylori* in exacerbating micronutrient deficiencies (vitamin B12, vitamin D, and iron) in Ezidi refugees resettling in rural Australia. This retrospective cohort study will inform potential changes to refugee health screening practices, addressing both infectious and nutritional challenges in this vulnerable population.

## Methods

### Participants

All available Ezidi refugee data from the Armidale Medical Practice and the Armidale Hospital initial refugee health assessment were included in this clinical cohort study. Individuals underwent *H. pylori* testing only if they met specific criteria:

- Symptomatic individuals: those presenting with symptoms such as epigastric pain, fatigue, or bloating*.*
- Micronutrient-deficient individuals: those with low iron or vitamin B12 levels, as identified through routine blood tests.
- Family members of H. pylori-positive individuals: those tested due to a confirmed *H. pylori* infection in a household member.

This targeted approach aligns with real-world clinical decision-making and follows Australian guidelines; however, it limits generalisability, as it may overestimate the true prevalence of *H. pylori* in the broader Ezidi refugee population.

### Data Collection

Patient data were extracted from two healthcare settings serving Ezidi refugees in Armidale: the Armidale Medical Centre and the Armidale Hospital. The Armidale Hospital primarily conducted initial refugee health assessments upon arrival, while the Armidale Medical Centre provided ongoing general practice care, including further investigations and management for symptomatic individuals or those with more complex clinical needs. As such, *H. pylori* testing was more commonly initiated in the general practice setting based on clinical presentation.

Data included demographic variables (date of birth, gender), *H. pylori* testing and treatment details, and levels of ferritin, vitamin D, and vitamin B12 (pre- and post-treatment (At least 6 weeks after the *H. pylori* therapy, including clarithromycin, amoxicillin, and esomeprazole triple therapy for 7–14 days per Australian guidelines). Following extraction, the data were de-identified, reviewed for completeness and accuracy, and analysed. This protocol was approved by the University of New England Ethical Committee (HE23-144). As this was a retrospective audit of de-identified data, the requirement for informed consent was waived in accordance with the National Statement on Ethical Conduct in Human Research (Australia, 2007). Prospective studies could benefit from the inclusion of a comparison group to better understand the relationship between *H. pylori* infection and micronutrient deficiencies.

### Measures

Three different commercial pathology laboratories within the Armidale region conducted blood tests for each micronutrient deficiency at Armidale Medical Centre. The blood tests at Armidale Hospital were collected at the onsite pathology lab and transported to Sydney for analysis. All laboratories measured vitamin D concentration using the Diasorin method (imprecision rate of 5.2% or less)(19). Chemiluminescence and the Siemens method (detection limit: 125 pg/mL–2.2 pg/mL) were used for vitamin B12 levels (20). Ferritin concentration was measured using chemiluminescence microparticle immunoassay (CMIA) (sensitivity ≤ 1.0 ng/mL (21) and the Siemens method (detection limit ≤ 1.0 ng/mL)(22). These methods are consistent with current Australian clinical standards and provided reliable data.

According to clinical Australian guidelines and for practical considerations*, H. pylori* infection was identified by urea breath testing (UBT) using the Tri-Med PYtest kit for patients over 14 years old, and stool PCR for those under 14 or when UBT was unsuitable, such as in cases of intellectual disability. Patients under 3 were not tested. UBT’s sensitivity and specificity are 96% and 93%, respectively (23) in adults, and 96.6% and 97.7% (24) in children over 6 years. Stool PCR shows a sensitivity of 92% and specificity of 96%(25).

Deficiency criteria for the study were ferritin < 30 µg/L, vitamin D < 50 nmol/L, and total serum B12 < 138 pmol/L in conjunction with low Active vitamin B12 level. B12 testing was performed on total serum B12 levels between 139–250 pmol/L, and Active B12 levels < 50 pmol/L were considered deficient. Post-treatment testing was conducted during follow-up appointments after the appropriate treatment per Australian therapeutic guidelines (oral or parenteral supplementation). In some instances, comprehensive testing of all variables didn’t occur. Many patients were lost to follow-up, primarily due to patient relocation. For the missing variables, patients were excluded from the analysis.

### Data Analysis

The Jamovi software was used to calculate the prevalence rates of *H. pylori* infection and micronutrient deficiencies. Both the chi-squared test of association and Fisher’s Exact test were used to evaluate the association between *H. pylori* infection and micronutrient deficiencies in the clinical cohort and in a subgroup of participants < 16 years of age.

## Results

### Patients *H. pylori* Testing

A total of 260 Ezidi refugee patients (female (n = 156), male (n = 104) of all ages (ranging from 6 months to 75 years) in the Armidale Medical Practice database up until Jan 2024 were included for analysis.

**Fig. 1:**
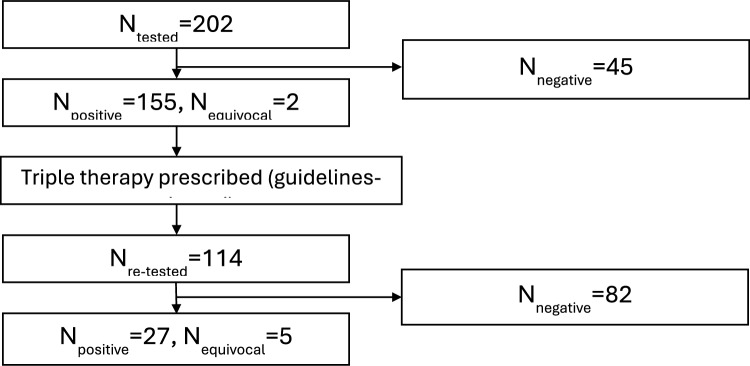
Flow diagram of *H. pylori* testing conducted at Armidale Medical Centre

As summarised in Fig. 1, 202 patients underwent *H. pylori* testing: 155 patients tested positive, 45 tested negative, 2 had equivocal results and 58 were not tested. Following *H. pylori* guideline-based treatment, 114 patients were retested: 27 remained positive, 82 tested negative, and 5 had equivocal results (Indeterminate *H. pylori* test results). Given that 157 patients had positive or equivocal results on initial testing, there is a follow-up gap for 43 patients who possibly relocated or failed to follow up. The “equivocal results” indicate “indeterminate *H. pylori* test results”. These cases involved borderline breath test results that could not definitively confirm infection status.

### Prevalence of Vitamin B12, Vitamin D, and Ferritin Before and After Treatment

Ferritin levels were tested in 230 patients, with 35.2% found to be deficient. After treatment, 125 were retested, and 24% remained deficient (Table 1).

**Table 1:** Prevalence of iron, vitamin D and B12 deficiencies

Organisation	Context of testing	Micronutrient deficiency	Counts	% of total
*Iron (ferritin) deficiency*				
Armidale Medical Centre	Pre-treatment	Yes	81	35.2
		No	149	64.8
	Post-treatment	Yes	30	24.0
		No	95	76.0
Armidale Hospital	Initial Refugee Health Assessment	Yes	166	41.9
		No	230	58.1
*Vitamin D deficiency*				
Armidale Medical Centre	Pre-treatment	Yes	183	79.6
		No	47	20.4
	Post-treatment	Yes	78	69.6
		No	34	30.4
Armidale Hospital	Initial Refugee Health Assessment	Yes	327	82.6
		No	69	17.4
*Vitamin B12 deficiency*				
Armidale Medical Centre	Pre-treatment	Yes	113	50.7
		No	110	49.3
	Post-treatment	Yes	19	17.0
		No	93	83.0
Armidale Hospital	Initial Refugee Health Assessment	Yes	225	58.0
		No	163	42.0

Of the 260 patients, 230 were tested for vitamin D deficiency, and 183 (79.6%) were found to be deficient. After supplementation, 112 were retested, with 78 (69.6%) still deficient (Table 1).

For vitamin B12 levels, 223 patients were tested, and 113 (50.7%) were found to be deficient. After receiving dietary advice and parenteral supplementation, 112 were retested, and 19 (17.0%) remained deficient (Table 1).

Separately, data from Armidale Hospital included 507 patients. Of these, ferritin levels were tested in 396 patients, with 166 (41.9%) found to be iron deficient. The same 396 were also tested for vitamin D deficiency, and 327 (82.6%) were found to be deficient. For vitamin B12, 388 were tested, with 225 (58.0%) found to be deficient.

Further focus study was obtained from data extracted from Armidale Medical Centre refugee patients:

### Association Between Iron Deficiency and *H. pylori* Status

Serum ferritin was assessed in 197 of 202 patients tested for H. pylori before treatment. Among 153 *H. pylori*-positive patients, 53 (34.6%) were iron deficient. Among 42 *H. pylori*-negative patients, 14 (33.3%) were iron deficient (Table 2). Two patients had equivocal results, one was iron deficient, and the other was not. Statistical analysis showed no significant relationship between *H. pylori* status and iron deficiency both in the clinical cohort and the < 16-year-old subgroup (p-value = 0.887 on chi-squared test of association and 1.00 on Fisher’s exact test (Table 2); p value = 0.553 and 0.596 on chi-squared test and Fisher’s exact test respectively (Table 3)).

**Table 2:** *H. pylori* status and iron deficiency before and after treatment

	Iron deficient (pre-treatment)	Iron deficient (post-treatment)				
*H. pylori* test result	Yes	No	Total	Yes	No	Total
Positive	53	100	153	7	10	17
Negative	14	28	42	13	44	57
Equivocal	1	1	2	2	2	4
Total	68	129	197	22	56	78

Tests of association	Pre-treatment		Post-treatment	
	Value	p	Value	p
χ^2^	0.239	0.887	3.17	0.205
Fisher's exact test		1		0.181

**Table 3:** *H. pylori* status and iron deficiency before and after treatment in children < 16 years of age

Children < 16 years	Iron deficient (pre-treatment)			Iron deficient (post-treatment)		
*H. pylori* test result	Yes	No	Total	Yes	No	Total
Positive	15	41	56	5	2	7
Negative	8	16	24	3	14	17
Total	23	57	80	8	16	24

Tests of association	Pre-treatment		Post-treatment	
	Value	p	Value	p
χ^2^	0.352	0.553	6.45	0.011
Fisher's exact test		0.596		0.021

After *H. pylori* treatment, serum ferritin was assessed in 78 patients. Among 17 patients who remained *H. pylori* positive, 7 (41.2%) had persistent iron deficiency. Among 57 patients who tested negative for *H. pylori* post-treatment, 13 (22.8%) remained iron deficient. Four patients had equivocal results, e.g. where the test was inconclusive, with two being iron deficient. The association between post-treatment *H. pylori* status and iron deficiency was not statistically significant (p-value = 0.181 on Fisher’s exact test). In the subgroup < 16-years-old, among the 7 patients that remained *H. pylori* positive post-treatment, 5 (71.4%) remained iron deficient. Among the 17 patients who tested negative for *H. pylori* post-treatment, 3 (17.6%) remained iron deficient (Table 2). Statistical analysis revealed a significant relationship between post-treatment *H. pylori* status and iron deficiency (p-value = 0.021 on Fisher’s exact test) (Table 3).

### Association Between Vitamin D Deficiency and *H. pylori* Status

Serum vitamin D was assessed in 190 of 202 patients before *H. pylori* treatment. Among 148 *H. pylori*-positive patients, 117 (79.1%) were vitamin D deficient. Of the 40 *H. pylori*-negative patients, 32 (80%) were vitamin D deficient. Two patients had equivocal results, with one being vitamin D deficient. Statistical analysis showed no significant relationship between *H. pylori* status and vitamin D deficiency in both the clinical cohort and < 16-year-old subgroup (p-value = 0.554, 0.432 respectively) (Tables 4, 5).

**Table 4:** *H. pylori* status and vitamin D deficiency before and after treatment

	Vitamin D deficient (pre-treatment)	Vitamin D deficient (post-treatment)				
Initial *H. pylori* test result	Yes	No	Total	Yes	No	Total
Positive	117	31	148	13	4	17
Negative	32	8	40	37	18	55
Equivocal	1	1	2	3	1	4
Total	150	40	190	53	23	76

Tests of association	Pre-treatment		Post-treatment	
	Value	p	Value	p
χ^2^	1.04	0.596	0.576	0.75
Fisher's exact test		0.554		0.815

**Table 5:** *H. pylori* status and Vitamin D deficiency before and after treatment in children < 16 years of age

Children < 16 years	Vitamin D deficient (pre-treatment)	Vitamin D deficient (post-treatment)				
Initial *H. pylori* test result	Yes	No	Total	Yes	No	Total
Positive	33	20	53	6	1	7
Negative	17	6	23	7	8	15
Total	50	26	76	13	9	22

Tests of association	Pre-treatment		Post-treatment	
	Value	p	Value	p
χ^2^	0.967	0.325	3.01	0.083
Fisher's exact test		0.432		0.165

After *H. pylori* treatment, serum vitamin D was assessed in 76 patients. Of the 17 patients who remained *H. pylori* positive, 13 (76.5%) were still vitamin D deficient. Among the 55 patients who tested negative for *H. pylori* post-treatment, 37 (66.7%) remained vitamin D deficient. Four patients had equivocal results, with three being vitamin D deficient. The relationship between post-treatment *H. pylori* status and vitamin D deficiency was not statistically significant in both groups (p-value = 0.815, 0.165 respectively) (Tables 4, 5).

### Association Between Vitamin B12 Deficiency and *H. pylori* Status

Serum vitamin B12 was assessed in 190 of 202 patients before *H. pylori* treatment. Among 147 *H. pylori*-positive patients, 70 (47.6%) were vitamin B12 deficient. Of the 41 *H. pylori*-negative patients, 27 (65.9%) were vitamin B12 deficient. Two patients had equivocal results, both of whom were vitamin B12 deficient. Statistical analysis revealed a significant relationship between H. pylori status and vitamin B12 deficiency in the clinical cohort (p-value = 0.032) (Table 6) but not in the < 16-year-old subgroup (p-value = 0.142) (TTable 5).

**Table 6:** *H. pylori* status and Vitamin B12 deficiency before and after treatment

	Vitamin B12 deficient (pre-treatment)	Vit B12 deficient (post-treatment)				
Initial *H. pylori* test result	Yes	No	Total	Yes	No	Total
Positive	70	77	147	1	15	16
Negative	27	14	41	11	43	54
Equivocal	2	0	2	0	4	4
Total	99	91	190	12	62	74

Tests of association	Pre-treatment		Post-treatment	
	Value	p	Value	p
χ^2^	6.13	0.047	2.63	0.269
Fisher's exact test		0.032		0.425

After *H. pylori* treatment, serum vitamin B12 was assessed in 74 patients. Among 16 patients who remained H. pylori positive, 1 (6.7%) remained vitamin B12 deficient, and 15 (93.3%) were not. Among 54 patients who tested negative for *H. pylori* post-treatment, 11 (20.4%) remained vitamin B12 deficient, and 43 (79.6%) were not. Four patients had equivocal results, none of whom were vitamin B12 deficient. The relationship between vitamin B12 deficiency and *H. pylori* status post-treatment was not statistically significant in both groups (p-value = 0.425, 1.000 respectively) (Tables 6, 7).

**Table 7:** *H. pylori* status and Vitamin B12 deficiency before and after treatment in children < 16 years of age

Children < 16 years	Vitamin B12 deficient (pre-treatment)	Vit B12 deficient (post-treatment)				
Initial *H. pylori* test result	Yes	No	Total	Yes	No	Total
Positive	22	29	51	1	6	7
Negative	15	9	24	3	12	15
Total	37	38	75	4	18	22

Tests of association	Pre-treatment		Post-treatment	
	Value	p	Value	P
χ^2^	2.45	0.118	0.105	0.746
Fisher's exact test		0.142		1.000

## Discussion

### Prevalence of *H. pylori* Infection

The prevalence of *H. pylori* infection in Ezidi refugees was 76.7%. This is significantly higher than the global rates (43.9% in adults, 35.1% in children) (26)and higher than the rates reported in Iraq (54.2% in adults, 37.1% in children)(26). This is consistent with other studies reporting *H. pylori* prevalence between 72 – 93% in refugees and immigrants in Western countries(27). However, it exceeds the 21.5% prevalence observed among newly arrived refugees in South Australia(15). The high rates of H. pylori infection among Ezidi refugees may be linked to environmental and social factors such as overcrowding, poor sanitation, large family sizes, and food sharing, conditions that facilitate transmission. These issues are common in refugee settings and have been associated with increased H. pylori risk in other refugee populations, including those in South Australia(15). Limited access to healthcare in camps further compounds these risks. Further research is needed to disentangles these overlapping factors.

### Iron Deficiency and *H. pylori* Infection

Iron deficiency was similar in both study samples (41.9% at Armidale Hospital and 35.2% at Armidale Medical Centre). This is notably higher than the Australian general population (35, 36). Overall, our study found no statistically significant association between *H. pylori* and iron deficiency as measured by serum ferritin, in contrast to several studies that report a significant association between *H. pylori* infection and iron deficiency(14, 28–30), as well as improvements in serum ferritin following *H. pylori* eradication(14, 28–30). However, a subgroup analysis of children < 16-years-old demonstrated a statistically significant association between persistent *H. pylori* infection post eradication therapy and persistent iron deficiency. Likewise, the association between a negative *H. pylori* status post-treatment and absence of iron deficiency was statistically significant. This result suggests that successful *H. pylori* eradication may improve iron status in iron deficient children, however, this was seen in a small sample size. Similar findings have been observed in other studies examining the relationship between *H. pylori* and iron deficiency in children (13, 31, 32). However, persistent iron deficiency or anaemia has been observed for up to two years post *H. pylori* eradication(33), raising the possibility of persisting gastritis impacting iron levels after *H pylori* eradication.

Kishore et al. reported higher serum ferritin levels in *H. pylori-*infected individuals compared to the uninfected group(34). A physiological immune defence mechanism, which increases iron storage and ferritin levels during infectious diseases and inflammation to limit bacterial access to needed iron for growth, may explain this(35). Since we could only rely on measured serum ferritin levels to assess iron deficiency, this mechanism may explain our non-statistically significant results. It is possible that the inflammatory response caused by *H. pylori* infection led to normal or increased serum ferritin levels in our study cohort, masking an underlying iron deficiency. Future research investigating the impact of *H. pylori* on iron deficiency and iron deficiency anaemia should include additional parameters such as a haemoglobin concentration, mean corpuscular volume, mean corpuscular haemoglobin, total iron binding capacity, ferritin, transferrin, and transferrin saturation.

### Vitamin B12 Deficiency and *H. pylori* Infection

Vitamin B12 deficiency was high in both samples (58% at Armidale Hospital, 50.7% at Armidale Medical Centre), exceeding the rates seen in the general Australian population (0.9–2.8%)(36). These rates are even higher than vulnerable populations such as the elderly (14%) and vegans (26%)(37, 38). In comparison, a study that also examined refugees from a similar Middle Eastern background revealed a 16.5% prevalence rate(39).

Interestingly, our study found that Ezidi refugees without *H. pylori* infection were more likely to be vitamin B12 deficient than those with *H. pylori* infection, both before and after eradication therapy. This suggests that *H. pylori* may have a minor protective effect against vitamin B12 deficiency. These findings are inconsistent with previous studies, some of which report no association between *H. pylori* and vitamin B12 levels(10), while others identify *H. pylor*i infection as a significant risk factor for vitamin B12 deficiency(10, 40, 41).

*H. pylori* is known to cause atrophic gastritis and destruction of gastric parietal cells(41), leading to impaired stomach acidification and reduced intrinsic factor secretion, both of which are required for vitamin B12 transport and absorption(10, 42). The higher rate of vitamin B12 deficiency in *H. pylori-*negative individuals in our study is surprising, as previous research findings align more with vitamin B12 malabsorption mechanisms(8, 43, 44). To explore potential autoimmune causes, blood tests for intrinsic factor and parietal cell antibodies were ordered for all B12-deficient refugees, with only one patient returning a positive result for gastric parietal cell antibodies.

Lifestyle factors and comorbid medical conditions may have influenced the vitamin B12 levels observed, but establishing an association between those components is beyond the scope of this study. Among patients who were both *H. pylori* positive and vitamin B12 deficient, we found a rate of 47.6%, which is close to rates reported in previous studies(8, 40, 44). Additionally, we observed a decrease in the prevalence of vitamin B12 deficiency from 52.1% to 16.2% after treatment. However, there was a significant number of missing follow-up data, which does introduce some bias.

### Vitamin D Deficiency and *H. pylori* Infection

Vitamin D deficiency was similar in both study samples (79.6% at Armidale Medical Centre, 82.6% at Armidale Hospital), closely matching a previously reported rate of 89% in Middle Eastern refugees(45). Nevertheless, this is significantly higher than the approximately 23% prevalence reported in the Australian population (46). The high prevalence observed in our study may attributed to factors such as the wearing of conservative clothing (scarves or hijabs) and a cultural aversion to tanning.

Our study found no statistically significant link between *H. pylori* infection and vitamin D deficiency, either pre- or post-treatment. As of today, the relationship between these two factors remains unclear(12). However, some studies suggest that vitamin D may act as a protective factor against *H. pylori* infection, potentially improving the success of eradication therapy (12, 47) but further studies are required to establish a connection between H. pylori infection, vitamin D deficiency, supplementation, and its impact on *H. pylori* treatment(48).

### Limitations

Several limitations must be acknowledged. The small sample size of Ezidi refugees in Armidale reduces the study's statistical power. The data extraction process using Best Practice software compared to manual extraction may have introduced inconsistencies, however the data were double-checked by two collectors. The selective nature of pathology testing, based on clinical suspicion, introduces sampling bias, potentially inflating prevalence rates. Additionally, the use of multiple commercial laboratories for pathology testing may have caused minor detection bias. However, this bias is likely limited, as deficiency rates were similar between the Armidale Medical Centre and the Armidale Hospital.

Due to the nature of the data available, the inclusion of a control group was not feasible in this study.

Variable treatment durations and inconsistent follow-up times may also influence post-treatment prevalence rates due to bias. Moreover, data on treatment eradication outcomes, such as *H. pylori* eradication, were not available. This is a notable limitation, as previous research in other immigrant populations suggests eradication rates are often lower than the general population. We recommend that future studies specifically collect and evaluate these outcomes.

The study did not fully account for confounding factors such as age, comorbidities, and diet, which could have affected the results. For example, polyphenol-containing beverages like tea and coffee significantly reduce iron absorption, and conditions like coeliac disease are known to cause micronutrient deficiencies due to malabsorption. Finally, the retrospective nature of the study, relying on past records, may have resulted in incomplete data.

Inclusion of additional micronutrient markers would have strengthened our analysis. However, these tests were not routinely conducted in the clinical setting for this cohort as per Australian clinical guidelines, and were therefore not available for retrospective analysis.

### Contribution to the Literature

This is the first study to asses and compare the prevalence of *H. pylori*, iron deficiency, vitamin B12 deficiency, and vitamin D deficiency among Ezidi refugees in Armidale, a rural Australian town selected for regional settlement through a specialised program (49). The study found no significant correlation between *H pylori* infection, iron and vitamin D deficiencies, but it did observe a significant association between vitamin B12 deficiency and *H. pylori*-negative individuals. A significant association of post-treatment *H. pylori* infection status and iron deficiency was observed in children in a small sample size. The overall relationship between *H. pylori* infection and micronutrient deficiencies remains unclear, as previous studies have reported mixed and inconsistent results. This study emphasises the need for further research to clarify these associations and supports the potential for standardised screening protocols for Ezidi refugees.

## Data Availability

Unidentified data will be provided if needed, it is saved at Armidale Medical Centre patient confidential file.
